# Advantages of echocardiography in the emergency room in front of a young patient with palpitations

**DOI:** 10.1186/2036-7902-7-S1-A19

**Published:** 2015-03-09

**Authors:** AA Oviedo-García, M Algaba-Montes

**Affiliations:** 1 Emergency Department, Valme Hospital, Seville, Spain

## Background

Apical hypertrophic cardiomyopathy (AHM) is a variant of hypertrophic cardiomyopathy, involving nearly exclusively apex. It is very common among Asian patients, predominantly in Japanese, which is considered relatively benign condition. However, severe clinical manifestations, including sudden cardiac death, severe arrhythmias and apical infarction have been described in case reports. The electrocardiographic changes (giant negative T waves ) and associated symptoms (chest pain, palpitations, dyspnea…) often present as acute coronary síndromes. AHM diagnosis is based on the demonstration of myocardial hypertrophy in the apical region of the left ventricle, usually by echocardiography with classical image "ace of spades", although in many cases the use of contrast necessary.

## Objective

We present a case of young patient admited at ER with palpitations.

## Patients and methods

The most frequent morbid events in Eriksson et al study of AHM were atrial fibrillation (AF), probably related to left atrial enlargement and impaired LV relaxation. It is prudent to also closely examine the heart on bedside emergency echocardiography looking for the presence of left atrial enlargement.

## Results

37 year old male, with no significant medical history, was admitted to the ER by palpitations. The electrocardiogram showed AF with deep, negative T-waves in leads V3-V6. Bedside emergency echocardiography (BEE) initially performed to look for left atrial enlargement, revealed apical hypertrophy, with apical cavity obliteration during systole. These findings were confirmed by contrast ecocardiography. The patient was diagnosed with AHM (Yamaguchi's syndrome) and started on beta-blocker therapy.

## Conclusion

In this case, BEE helped to identify an AHM. It was the findings on emergency ultrasound, performed and interpreted by EPs, that helped to identify the correct diagnosis and prompted the appropriate consultations to cardiologist, with a final diagnostic of AHM.

## Informed consent

The study was conducted in accordance with the ethical standards dictated by applicable law. Informed consent was obtained from each owner to enrolment in the study and to the inclusion in this article of information that could potentially lead to their identification.
